# Estrogen mimicking effects of xenobiotics in fish

**DOI:** 10.1186/1751-0147-54-S1-S12

**Published:** 2012-02-24

**Authors:** Poul Bjerregaard

**Affiliations:** 1 Institute of Biology, University of Southern Denmark, Campusvej 55, DK-5230 Odense M, Denmark

## 

Sex hormones produced in humans and livestock are excreted in the urine. Naturally produced and synthetic contraceptive pill estrogens may pass low-quality waste water processing plants and feminize male fish in streams and coastal areas downstream such discharges [1,2]. High-quality processing of the wastewater with tertiary treatment, nutrient removal and sufficient water and sludge retention times basically removes the problem [3,4].

The feminization of male fish downstream waste water discharges has mainly been demonstrated by the presence of elevated levels of female yolk protein (vitellogenin) in the blood of male or juvenile fish or eggs (or oocytes) in the testes (intersex) [5]. Both of these phenomena are used as biomarkers for estrogenic effects and as always in the use of biomarkers, it is important to define the natural background level in an uncontaminated environment.

The first investigations on intersex in roach showed that male fish in areas not directly affected by wastewater discharges had intersex percentages between 5 and 11% [1,6], suggesting that there might be a low, natural occurrence of this phenomenon. Recent investigations [7] in pristine areas do, however, show no intersex among the males, indicating that the natural background level of intersex roach may actually be zero.

Plasma levels in male or juvenile fish have been used as an efficient biomarker to detect estrogenic contamina-tion in both freshwater and marine areas and a number of studies [e.g. [8,9]] have presented vitellogenin levels that are markedly and unambiguously elevated - maybe several orders of magnitude - compared to background levels. Less effort has been devoted to defining when less severe increases in plasma vitellogenin levels actually indicate a difference relative to unaffected background levels [10]. The occurrence of intersex in roach and elevated vitellogenin levels in areas not directly affected by discharges from sewage treatment plants suggests that there may be other inputs of estrogenic activity to the aquatic environment and, in fact, several possibilities – some of them associated with intensive, modern agriculture - exist.

Estrogens have been found to leach to the water draining from fields treated with pig manure [11], which generally has a high content of especially estrone and estrogenic activity has been found in streams in connection with dairy cattle farms [12].

Leguminous plants - such as clover, peas, lupine and alfalfa used as nitrogen binding crop in e.g. organic farming - produce phytoestrogens which may leach to - and are detected in - the freshwater environment [13] – most often in the ng/l range. Some phytoestrogens induce vitellogenin synthesis in the low µg/l range [14].

Houses in the open land are not all connected to central sewage treatment plants and simple waste water processing measures such as septic tanks may be employed. Septic tanks do a very poor job removing estrogens from wastewater and estrogenic activities 20 times higher than needed to feminize brown trout have been detected in discharges from such systems [15].

Recently, exposure to 17β-estradiol and octylphenol has been shown to cause malformations in embryos of viviparous eelpout at fairly high – but still environmentally realistic concentrations [16].
